# Outcome and Prognosis of Patients With Lupus Nephritis Submitted to Renal Transplantation

**DOI:** 10.1038/s41598-019-48070-y

**Published:** 2019-08-12

**Authors:** Bruna Coelho Albuquerque, Vivian Brito Salles, Rodrigo Dib de Paulo Tajra, Carlos Ewerton Maia Rodrigues

**Affiliations:** 1 Master’s Degree in Medical Sciences, Postgraduate Program, University of Fortaleza (Unifor), Fortaleza, Brazil; 2 Medical student at the University of Fortaleza (Unifor), Fortaleza, Brazil; 3 Medical student at the Federal University of Ceará, Fortaleza, Brazil; 4 Professor of Medical Sciences, Postgraduate Program, University of Fortaleza (Unifor) and Professor at the Federal University of Ceará, Fortaleza, Brazil

**Keywords:** Lupus nephritis, Lupus nephritis

## Abstract

This stydy aimed to evaluate the epidemiological and clinical profile and outcome of patients with lupus nephritis (LN) submitted to renal transplantation. Retrospective cohort study based on the records of 35 LN patients submitted to renal transplantation at a single center in Brazil between July 1996 and May 2016. The Kaplan-Meier method was used to estimate 6-month, 1-year and 5-year graft survival. The sample included 38 transplantations (3 of which retransplantations). The mean age at the time of SLE diagnosis was 23.7 ± 9.0 years. Most patients were female (94.7%) and 68.4% were non-Caucasian. Twenty-two (57.9%) underwent renal biopsy prior to transplantation. The mean time from SLE diagnosis to transplantation was 10.3 ± 6.4 years. The mean pre-transplantation dialysis time was 3.8 ± 3.7 years. The grafts came from living related (n = 11) or deceased (n = 27) donors. Three (7.9%) patients experienced acute rejection in the first year. Graft and patient survival rates were, respectively, 97.1% and 100% at 6 months, 84.9% and 96.9% at 1 year, and 76.3% and 92.5% at 5 years. One (2.6%) patient had SLE recurrence. Venous thrombosis (*p* = 0.017) and antiphospholipid syndrome (APS) (*p* = 0.036) were more prevalent in patients with graft loss. In our cohort of LN patients submitted to renal transplantation, the 5-year survival rate was high, and APS was an important predictor of poor renal outcome (graft loss).

## Introduction

Lupus nephritis (LN) is an important cause of morbidity and mortality due to the possibility of progression to renal failure and/or treatment-related complications. It is diagnosed in approximately 37% to 45% of patients with systemic lupus erythematosus (SLE) at some point during the course of the disease^1^.

The 5-year survival rate is significantly lower for SLE patients with than without LN. Thus, 10–30% of SLE patients with LN develop end-stage chronic kidney disease (CKD) within 15 years of LN diagnosis, despite aggressive treatment^2^.

Renal transplantation is a viable treatment option for patients with end-stage CKD and LN^3–6^, and the risk of graft failure is similar in renal transplantation patients with and without SLE^5,6^. Moreover, the rates of LN-related complications and recurrence are low^7^.

Studies conducted in Brazil have shown good levels of 5-year renal graft survival (81–91%)^8–10^. However, little is known about the prognosis of LN patients submitted to renal transplantation. The purpose of this study was therefore to draw a sociodemographic, clinical and laboratory profile of a cohort of Brazilian LN patients submitted to renal transplantation and evaluate factors predictive of renal graft and patient survival.

## Materials and Methods

### Study approval

This retrospective cohort study was carried out in a single transplantation center in Northeastern Brazil. Submitted through an online national research database (*Plataforma Brasil*), the study protocol was approved by the Research Ethics Committee of Hospital Geral de Fortaleza (HGF) and filed under entry #1408227. All patients gave their written informed consent. In the case of death or loss to follow-up, the consent form was required to be signed by the next of kin or by a legal representative. The methods used in this study were carried out in accordance with the approved protocols and guidelines. No organs/tissues were procured from prisoners and all transplantations were performed at HGF.

### Patients

The sample consisted of LN patients of all ages and both genders submitted to kidney transplantation at HGF between July 1996 and May 2016. During this period, 1861 kidney transplantations were performed for a variety of medical conditions, 38 of which in patients with LN. Since three LN patients underwent transplantation more than once, our sample represented 2% (38/1861) of all transplantations. All 35 LN patients were included in the sample and met the criteria of the American College of Rheumatology (ACR) for SLE^11^. LN was diagnosed by renal biopsy or based on the presence of persistent proteinuria (≥0.5 g/24 hours, or >3 + ) with dysmorphic glomerular hematuria and/or cell cylinders^12^.

### Data collection

Prior to transplantation, information was collected on sex, ethnicity and age (whole years) at the time of SLE diagnosis. Clinical information was collected on renal biopsy for the diagnosis of LN, SDI score (Systemic Lupus International Collaborating Clinics/American College of Rheumatology damage index), systemic arterial hypertension, diabetes mellitus, venous thrombosis (documented deep vein thrombosis and/or pulmonary embolism), antiphospholipid syndrome (APS), positive serology for hepatitis B, hepatitis C and HIV, miscarriages, smoking, body mass index (BMI) and retransplantation.

Patients who reported smoking within 6 months of the evaluation were classified as smokers^13^, but smoking load was not taken into account. APS was classified as arterial or venous thrombosis and/or obstetric morbidity (one or more births of normal neonates before the 34th week of gestation, one or more unexplained deaths of normal foetuses at or beyond the 10th week of gestation, or three or more unexplained spontaneous abortions before the 10th week of gestation) in the presence of antiphospholipid antibodies (aPLs) on two or more occasions at least 12 weeks apart^14^. Weight (kg) and height (m) were used to calculate BMI (kg/m^2^). Patients were stratified according to the World Health Organization classification as normal weight (BMI >18.50 to 24.99), overweight (BMI ≥25 to 29.99) or obese (BMI ≥30)^15^. SLE cumulative damage was measured with the Systemic Lupus International Collaborating Clinics/American College of Rheumatology (ACR) Damage Index (SDI). Scores ranged from 0 to 47 (damage was considered present if the score was ≥1). The presence of irreversible and cumulative and/or present alterations for at least six months was defined as permanent damage^16^. Panel-reactive antibodies (PRAs) were evaluated to determine the presence and specificity of anti-HLA antibodies.

The following transplantation data were collected: age at transplantation, time from SLE diagnosis to renal transplantation, previous dialysis and time (years) of dialysis until transplantation, donor type (deceased/living related), human leukocyte antigen (HLA) system incompatibilities, delayed graft function rate, length (days) of hospital stay, induction and maintenance therapies, renal graft function, urinary protein, cytomegalovirus (CMV) and polyomavirus (BK) viremia, readmissions within one month after hospital discharge and their causes, acute rejection in the first year after transplantation, SLE recurrence, graft loss, and death.

HLA system incompatibility was defined as ≥3. Delayed graft function was defined as the need for dialysis in the first week after transplantation.

As for maintenance, we collected information on immunosuppressive therapy following induction therapy (medication used in the intraoperative period and first 7–10 days of in-hospital recovery). Renal graft function was proxied by the estimated glomerular filtration rate (eGFR) (simplified MDRD equation with four variables) at 6 months, 1 year and 5 years.

CMV and BK viremia was detected on quantitative real-time PCR. Acute rejection in the first year after transplantation was diagnosed clinically or by biopsy. Graft loss was defined as a return to the dialysis program, or retransplantation. LN recurrence was determined by clinical and laboratory parameters (worsening of renal function, changes in urinary sediment with hematic or protein cylinders, complement consumption and anti-DNA positivity) and confirmed by renal biopsy.

### Statistical analysis

Clinical and demographic parameters were expressed as mean values ± standard deviation (continuous variables) or frequencies and percentages (categorical variables). Fisher’s exact test (categorical variables) was used to compare patients with and without graft loss. Phi coefficient was used to evaluate the magnitude of the association between qualitative variables. Relationships were classified as strong (0.5–1.0), moderate (0.3–0.5) or weak (0.1–0.3). Kaplan-Meier charts were used to estimate 6-month, 1-year and 5-year graft survival. The level of statistical significance was set at 5% (*p* < 0.05) and all analyses were performed with the software IBM SPSS Statistics (IBM Corp., Armonk, NY, USA).

## Results

### Patient characteristics

Patients were predominantly female (94.7%) and non-Caucasian (68.4%). The mean age at the time of SLE diagnosis was 23.7 ± 9.0 years. Only 22 patients (57.9%) were submitted to renal biopsy before transplantation. The mean SDI score was 4.7 ± 1.2. Prior to renal transplantation, the following comorbidities were observed: systemic arterial hypertension (n = 27; 71.1%), diabetes mellitus (n = 2; 5.3%), thrombosis (n = 9; 23.7%) and APS (n = 4; 10.5%) (Table 1). Twenty-seven patients (71%) were submitted to induction therapy (pulse therapy with solumedrol for 3 days and cyclophosphamide once a month for 6 months). The drugs used for pre-transplantation LN maintenance therapy were prednisone (84.4%), cyclophosphamide (21.9%), azathioprine (12.5%) and mycophenolate (2,6%). No patient used immunobiological drugs (e.g., rituximab). The records of 6 patients (15.8%) contained no information on the treatment provided.

**Table 1:** Sociodemographic, clinical and laboratory characteristics of patients with lupus nephritis submitted to renal transplantation.

Characteristics	n (%)
Female sex, n (%)	36 (94.7)
Ethnicity	
Caucasians, n (%)	12 (31.6)
Non-Caucasians, n (%)	26 (68.4)
Age at SLE diagnosis, years	23.7 ± 9.0
Renal biopsy, n (%)	22 (57.9)
SDI score	4.7 ± 1.2
Arterial hypertension, n (%)	27 (71.1)
Diabetes mellitus, n (%)	2 (5.3)
Venous thrombosis, n (%)	9 (23.7)
APS, n (%)	4 (10.5)
Anti-HCV positivity, n (%)	4 (10.5)
HBsAg positivity, n (%)	—
HIV positivity, n (%)	—
Miscarriage, n (%)	6 (28.6)
Smoking	6 (15.8)
BMI, kg/m²	21.0 ± 4.0
Retransplantation, n (%)	3 (7.9)

The continuous variables were expressed as mean ± standard deviation and the categorical variables as frequencies and percentages. SLE = systemic lupus erythematosus; SDI = Systemic Lupus International Collaborating Clinics/American College of Rheumatology (ACR) Damage Index; APS = antiphospholipid syndrome; Anti-HCV = antibody against hepatitis C virus; HBsAg = surface antigen of hepatitis B virus; HIV = human immunodeficiency virus; BMI = body mass index.

### Disease progress in LN patients submitted to renal transplantation

The mean time of post-transplantation follow-up was 3.5 ± 2.0 years (22 patients were followed for 5 years). The mean age at transplantation was 32.8 ± 10.9 years. The mean time from SLE diagnosis to renal transplantation was 10.3 ± 6.4 years. Most patients (97.4%) underwent dialysis prior to transplantation (hemodialysis in all cases). The mean time of pre-transplantation dialysis was 3.9 ± 3.7 years. The grafts came from living related (n = 11) or deceased donors (n = 27). All the patients were submitted to induction therapy for renal transplantation: anti-thymocyte globulin (ATG) (55.2%), interleukin-2 receptor (IL2) antagonists (44.7%) and corticoids (89.4%). One retransplanted patient was first treated with IL2 antagonists, then with ATG, while two other patients used ATG in both procedures. The most commonly used post-transplantation maintenance immunosuppressants were tacrolimus (57.9%), mycophenolate sodium (52.6%) and everolimus (15.8%). The most frequent causes of hospital readmission were infection (n = 6; 66.7%) and clinical problems (graft dysfunction, renal biopsy or pulse therapy) (n = 3; 33.3%). Graft loss was due to SLE recurrence (n = 1), BK viremia (n = 2), acute rejection (n = 3), chronic rejection (n = 2) or postoperative surgical complications (n = 1) (Table 2).

**Table 2:** Evolution characteristics of patients with lupus nephritis submitted to renal transplantation.

Characteristics	Descriptive statistics
Age at transplantation, years	32.8 ± 10.9
Time of SLE diagnosis until transplantation, years	10.3 ± 6.4
Dialysis prior to transplantation, n (%)	37 (97.4)
Time of dialysis prior to transplantation, years	3.9 ± 3.7
Transplant donor, deceased/living related	27/11
Cold ischemia time, hours	17.5 ± 10.9
≥3 HLA incompatibilities, n (%)	30 (78.9)
PRA, n(%)	
PRA negative	20 (52,6)
PRA 1–49%	6 (15,8)
PRA >50%	6 (15,8)
Delayed graft function, n (%)	18 (47.4)
Length of hospital stay, days	16.7 ± 9.9
Maintenance immunosuppression therapy, n (%)	
Tacrolimus	22 (57.9)
Mycophenolate sodium	20 (52.6)
Everolimus	6 (15.8)
Sirolimus	1 (2.6)
Mycophenolate mofetil	4 (10.5)
Cyclosporine	2 (5.3)
Prednisone (mean dose 5 mg/day)	24 (63.2)
Renal graft function (eGFR)	
6 months	69.0 ± 25.6
1 year	67.7 ± 20.41
5 years	66.7 ± 18.25
Altered urinary protein levels, n(%)	
6 months	8 (21.1)
1 year	12 (31.6)
5 years	4 (10.5)
CMV viremia, n(%)	9 (23.7)
BK viremia, n(%)	2 (5.3)
Readmissions one month after hospital discharge	1.4 ± 0.8
Causes of readmissions in the 1st month, n(%)	
Surgical	—
Infectious	6 (66.7)
Immunological	3 (33.3)
Cardiovascular	—
Acute rejection in the 1st year, n(%)	3 (7.9)
SLE recurrence, n(%)	1 (2.6)
Loss of graft, n(%)	9 (23.7)
Death, n(%)	2 (5.3)

The continuous variables were expressed as mean ± standard deviation and the categorical variables as frequencies as percentages. eGFR=estimated glomerular filtration rate; SLE = systemic lupus erythematosus; HLA = human leucocyte antigen; PRA = panel reactive antibody; eGFR = estimated glomerular filtration rate, CMV = cytomegalovirus; BK (polyomavirus) viremia.

**Figure 1:**
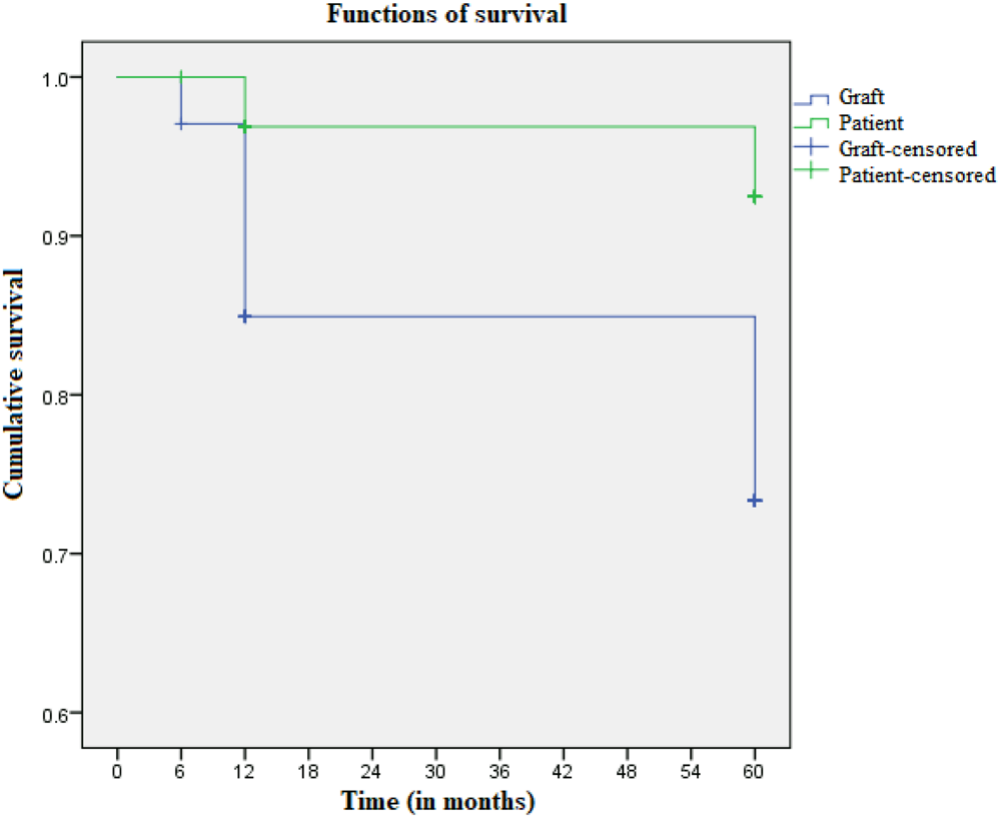
Kaplan-Meier curve showing patient and renal graft survival at 6 months, 1 year and 5 years.

Graft maintenance was observed for 29 recipients (76.3%). The 6-month, 1-year and 5-year graft and patient survival rates were, respectively, 97.1% and 100%, 84.9% and 96.9%, and 76.3% and 92.5% (Fig. 1).

### Comparative analysis of renal outcome

Patients with and without graft loss were compared with regard to hypertension, smoking, venous thrombosis, APS, positivity for hepatitis C, CMV viremia, BK viremia, and living related donor (information was incomplete for some patients). The analysis showed that venous thrombosis (5/8 [62.5] vs. 4/26 [15.4]; Phi coefficient = 0.45; *p* = 0.017 and APS (3/8 [37.5] vs. 1/25 [4.0]; Phi coefficient = 0.44; *p* = 0.036) were significantly more prevalent in patients with graft loss (Table 3).

**Table 3:** Comparative analysis of patients with lupus nephritis submitted to renal transplant with regard to renal outcome.

Variables (n%)	Graft loss (n = 9)	No graft loss (n = 29)	Phi coefficient	*p*-value*
Hypertension	5/8 (62.5)	22/27 (81.5)	0.190	0.346
Smoking	2/8 (25.0)	4/24 (16.7)	0.092	0.625
Anti-HCV	—	4 /29(13.8)	0.191	0.554
Venous thrombosis	5/8 (62.5)	4/26 (15.4)	0.453	0.017
APS	3/8 (37.5)	1/25 (4.0)	0.440	0.036
CMV viremia	—	9/24 (34.6)	0.318	0.149
BK viremia	2/8 (25.0)	—	0.447	0.056
Living related donor	3/9 (33.3)	8/29 (27.6)	0.054	1.000

*Fisher’s exact test; Anti-HCV = antibody against hepatitis C virus; APS = antiphospholipid syndrome; CMV = cytomegalovirus. BK = (polyomavirus). Level of statistical significance: p<0.05.

Nine patients had thrombosis (venous thrombosis n = 6; arterial thrombosis of the upper limbs n = 3) and four had APS. All these patients were treated with anticoagulants and/or prednisone (n = 8), cyclophosphamide (n = 2), azathioprine (n = 2), chloroquine (n = 1) and mycophenolate mofetil (n = 1). All were female, one was a smoker, one was submitted to retransplantation, and seven had hypertension. Two patients had a mean time from LN diagnosis to dialysis and from dialysis to transplantation of 5 and 3 years, respectively. All patients with thrombosis/APS had low levels of antibodies. Lupus anticoagulant and anticardiolipin antibody IgG was found in 3 patients with APS, anticardiolipin antibody IgM n = 2 and anti-β2glycoprotein-1 IgM (n = 1). At the time of transplantation, all patients with thrombosis/APS were submitted to an anticoagulation protocol (replacement of warfarin with low molecular weight heparin during the 7 days preceding transplantation). Heparin was stopped 24 hours before the surgical procedure and restarted 6 to 8 hours after the procedure. In most cases, warfarin was restarted 24 hours after transplantation.

## Discussion

In this study we evaluated the clinical and epidemiological characteristics, graft survival time, patient survival and its determinants, and recurrence of SLE in a cohort of LN patients submitted to renal transplantation at our institution between 1996 and 2016, and compared our findings with the national and international literature. To our knowledge, only three other studies on renal transplantation patients with SLE and LN have been conducted in Brazil^8–10^.

The long-term prognosis of renal transplantation patients with SLE is a matter of controversy, but some studies have shown patient survival to be similar in graft recipients with and without SLE^6,17–20^. During the 5-year follow-up period of this study, only 5.3% died, matching findings in the literature, including the three Brazilian studies evaluating renal transplantation patients with SLE and LN^8–10,21^.

**Figure 2:**
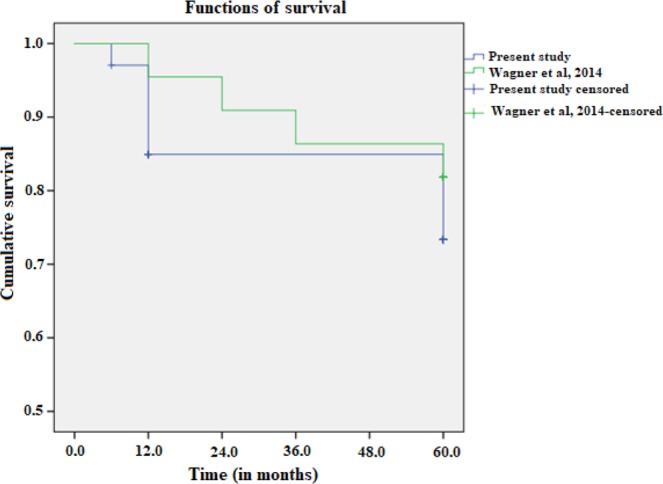
Kaplan-Meier graft survival curve comparing our results to those of a Brazilian series from 2014 (Wagner *et al*.)^10^.

Clinical response of SLE patients to transplantation depends on population, ethnicity, socioeconomic status, donor-related factors and LN recurrence^10^. A study analyzing the outcome and prognostic factors of renal transplantation in LN patients reported 1-year and 5-year graft survival rates of 93.9% and 81.5%, respectively, in agreement with the present study^4^. Moreover, our findings for clinical evolution (over 90% of the patients were alive at 5 years) are compatible with those of two other Brazilian studies based on cohorts sociodemographically similar to ours (*p* = 0.36 and *p* = 0.45, respectively)^9,10^ (Fig. 2).

Our results match thoseooooooooooooooooooooo of studies on different ethnicities^4,8–10,19,20,22–25^. Graft and patient survival was 76.3–100% and 67–100%, respectively^4,8–10,19,20,22–25^, and the main causes of graft loss were acute rejection, chronic allograft nephropathy and vascular thrombosis. Other observational cohorts of renal transplantation patients with LN from around the world (Brazil n = 14^9^, Brazil n = 18^10^, China n = 23^20^, Italy n = 33^22^, USA n = 1,170^23^, Korea n = 19^24^ and Mexico n = 74^25^) have yielded positive kidney outcomes similar to ours. Available evidence supports the notion that renal transplantation is a good treatment option for LN patients in dialysis, regardless of ethnicity.

In our cohort, no statistically significant association was observed between graft loss and the presence of hypertension or smoking―factors usually associated with decreased graft survival and cardiovascular complications^26,27^. In fact, a retrospective study including 2,886 LN patients submitted to renal transplantation (data from the United States Renal Data System and the United Network for Organ Sharing) concluded that cardiac events and vascular diseases were the leading causes of death in this patient population^28^. Norby *et al*.^29^ reported similar results (*p* = 0.018). This discrepancy in renal prognosis may be explained by the close follow-up and encouragement of blood pressure control and smoking cessation practiced at our center.

Despite the small sample size, our study suggests that venous thrombosis and APS had a negative influence on renal graft survival, probably by causing renal ischemia through the activation of platelets and fibrin in endothelial cells mediated by aPLs. Fuentes *et al*.^30^ reported vascular thrombosis as a cause of renal graft loss in 16.3% of patients with SLE submitted to transplantation, and Stone *et al*.^31^ reported clinical events associated with aPLs in 96 patients with SLE submitted to renal transplantation. Twenty-five of these (29.4%) had at least one positive test for aPLs, 10 (10.4%) died, 6 (6.25%) had deep vein thrombosis, and 4 (4.2%) had renal artery or vein thrombosis, suggesting a relation between graft failure and thrombotic disease associated with aPLs.

Another interesting finding was the high mean pre-transplantation dialysis time in our cohort (3.9 ± 3.7 years). In a Pakistanese study by Naveed *et al*.^32^, graft survival was greater in preemptive transplantation patients than in patients with pre-transplant dialysis. Likewise, Chinese researchers concluded that long-term pre-transplantation dialysis is associated with acute rejection and, consequently, poorer prognosis^33^.

A recent study concluded that LN patients undergoing dialysis should be referred for transplantation as early as possible, even in the presence of active SLE^33^. Although similar negative factors were relatively frequent in our sample, graft survival did not appear to be compromised, suggesting the influence of other as yet unidentified factors.

Winchester *et al*.^34^ argued that grafts from deceased donors are a better option for transplantation patients with SLE than grafts from living related donors due to the possibility of family inheritance through the HLA system. Grafts from relatives carry the same disease susceptibility genes and may increase the likelihood of SLE recurrence. Likewise, Cats *et al*.^35^ found 1-year graft survival rates to be significantly lower in recipients of grafts from living related donors. Nevertheless, in this study no difference in graft loss was observed between the two types of donors (*p* = 1.0).

Another surprising finding is the low frequency of acute rejection in our cohort (7.9%) when compared to the rates reported in three other Brazilian studies (18.2%, 42.8% and 55%, respectively)^8–10^. This may be explained by the low levels (15.8%) or absence (52.6%) of PRA, despite the higher degree of HLA mismatch. This hypothesis is supported by a study on African-American SLE patients reporting an apparent positive association between risk of kidney allograft rejection and grafts from deceased donors, with a higher degree of HLA mismatch and PRA^17^.

Viruses are the most important cause of infection, with significant mortality in renal transplantation recipients. The most commonly implicated pathogens are hepatitis C virus, CMV and BK virus^36^. In a retrospective study involving 1,624 patients submitted to renal transplantation, mortality and hospitalization rates were higher in hepatitis C-positive patients than in hepatitis C-negative patients^37^; likewise, the risk of graft loss was higher and renal function was worse in BK virus-positive patients than in BK-negative patients^38^. Lu *et al*.^39^ concluded that SLE patients had a higher prevalence of BK virus reactivation associated with a thrombocytopenic episode.

Approximately 80% of renal transplantation recipients develop BK viremia^38^. In the present study, no significant association (*p* = 0.056) was found between graft loss and BK viremia, and no patient with graft loss tested positive for CMV or hepatitis C, suggesting that BK viremia was not an important factor of graft loss in our cohort. This is probably due to the small sample size, and additional causes (aPLs, previous venous thrombosis, hypertension) should be investigated.

Despite the presence in our cohort of risk factors for SLE recurrence, such as female gender, pre-transplantation dialysis^40^, Latin-American ethnicity^41^, aPL^22^, and living donor grafts^29^, the rate of SLE recurrence was low (2.6%), matching the literature (2–11%)^22,23,41–43^. The standard immunosuppressive regimen our patients were submitted to (calcineurin inhibitors 76%, mycophenolate 63%, corticoids 63%) probably provided clinical protection against disease recurrence, associated with careful control of cardiovascular risk factors (hypertension, obesity) and close follow-up by multidisciplinary teams at specialized centers.

Our study was limited by the lack of a controlled post-transplantation treatment protocol, by the small sample size, and by the lack of an age and gender-matched control group of non-LN patients diagnosed with end-stage renal disease. Moreover, sociodemographic, clinical and treatment data may have been underreported, and not all important data (for example, progress of proteinuria) may have been analyzed due to the retrospective nature of the study. Finally, no information was collected on disease activity markers (complement C3/C4 and anti-dsDNA antibodies), the effect of histopathological findings on renal outcome, or post-transplantation thrombotic events.

Despite its limitations, our study yielded relevant results: LN patients submitted to renal transplantation displayed good 5-year survival rates and the presence of APS seemed to be a significant predictor of graft loss. Future longitudinal studies based on larger samples may shed light on the effect of disease status, associated conditions and the control of modifiable risk factors on the survival and quality of life of these patients.
